# Effect of COVID-19 Pandemic on Influenza Vaccination Intention: A Meta-Analysis and Systematic Review

**DOI:** 10.3390/vaccines10040606

**Published:** 2022-04-13

**Authors:** Gwyneth Kong, Nicole-Ann Lim, Yip Han Chin, Yvonne Peng Mei Ng, Zubair Amin

**Affiliations:** 1 Department of Medicine, Yong Loo Lin School of Medicine, National University of Singapore, Singapore 117597, Singapore; e0326129@u.nus.edu (G.K.); nicoleannlim@u.nus.edu (N.-A.L.); c.yiphan@u.nus.edu (Y.H.C.); 2 Department of Neonatology, Khoo Teck Puat-National University Children’s Medical Institute, National University Health System, Singapore 119228, Singapore; paeza@nus.edu.sg; 3 Department of Paediatrics, Yong Loo Lin School of Medicine, National University of Singapore, Singapore 117597, Singapore

**Keywords:** COVID-19, influenza, vaccination, vaccine hesitancy, meta-analysis

## Abstract

Poorer outcomes have been reported with COVID-19 and influenza coinfections. As the COVID-19 pandemic rages on, protection against influenza by vaccination is becoming increasingly important. This study examines how COVID-19 has influenced influenza vaccination intentions from a global perspective. A literature search was conducted on Embase, PubMed, and CNKI from 1 January 2019 to 31 December 2021 for articles reporting rates of influenza vaccination pre-COVID-19 (19/20 season), and intention and/or uptake of influenza vaccination post-COVID-19 (20/21 season). The changes in vaccination intention and reasons for changes were reported. Subgroup analyses were performed by region, gender, age, and occupation. Newcastle Ottawa Scale was used for quality assessment of the articles. Twenty-seven studies with 39,193 participants were included. Among 22 studies reporting intention to vaccinate in 20/21, there was increased intention to vaccinate (RR 1.50, 95% CI 1.32–1.69, *p* < 0.001) regardless of age, gender, and occupation. The remaining five studies reporting vaccination intention and uptake in 20/21 showed a similar increase (RR 1.68, 95%CI 1.20–2.36). Important determinants include historical vaccine acceptance, and perception of influenza severity and vaccine safety. The COVID-19 pandemic has increased intention to vaccinate against influenza internationally. The pandemic could be a window of opportunity to promote influenza vaccination and decrease vaccine hesitancy.

## 1. Introduction

According to the World Health Organization (WHO) Global Burden of Disease, lower respiratory tract infection (LRTI) is the fourth most important leading cause of death [1]. It accounted for up to 2.6 million deaths in 2019 and remains the world’s deadliest communicable disease [1]. Globally, influenza causes 389,000 (294,000–518,000) deaths annually, with two-thirds of deaths in adults above 65 years old [2]. Seasonal influenza contributes significantly to deaths from other causes (such as acute heart failure or ischemic heart disease) in vulnerable populations [3,4].

The impact of influenza on various populations has been a subject of great interest to many clinicians. The highest mortality rate was observed in Sub-Saharan Africa, Southeast Asia, and in the elderly above 75 years old [5]. Prognostic factors for mortality are affected by regional variations in baseline mortality, age structure, socio-demographic factors, and presence of co-existing health conditions [2]. Other established risk factors include children younger than 5 years [6], immunosuppressive conditions [6], obesity [7], and pregnancy [8].

Different strategies have been developed in the battle against influenza epidemics with vaccination as the cornerstone. Many countries have declared influenza vaccination as a priority healthcare goal [9], as vaccination is cost-effective [10,11] and efficient [12] in preventing influenza-associated morbidities. However, influenza vaccination uptakes are still not achieving set targets [13,14]. A study in 11 European countries demonstrated that gender, age, presence of chronic illness, household income, size of household, educational level, and population size of living residence, contribute to differences in influenza vaccination rates [15]. Healthcare workers in Asia were reported to have better vaccination rates than those in America [16].

The COVID-19 pandemic has disrupted the provision of healthcare services across the globe. Overlap of the 2019/2020 Northern Hemisphere and 2020 Southern Hemisphere seasonal influenza with COVID-19 has resulted in increasing prevalence of coinfections, leading to poorer outcomes and excess mortality [17]. Recent studies have postulated that influenza vaccination is associated with lower SARS-CoV-2 seroprevalence, hospitalizations, intensive care unit admissions, and deaths from COVID-19 [18]. Influenza vaccination can thus reduce the strain on healthcare resources caused by the COVID-19 pandemic by reducing the burden of influenza disease and improving the differentiation between influenza and COVID-19—two diseases with similar signs and symptoms [19].

Therefore, there is an urgent need to examine the impact of the COVID-19 pandemic on influenza vaccination from a global perspective. We aim to determine how the COVID-19 pandemic has influenced the intention to vaccinate against influenza virus across the globe. We compared vaccination rates for the 2019/2020 influenza vaccine with intention or actual update of the 2020/2021 influenza vaccine within the same group of individuals in the study cohort and examined reasons for this change. Our secondary aim is to determine variations in intention to vaccinate based on geographical regions, gender, age, and occupation, as well as the factors influencing this change in attitudes.

## 2. Materials and Methods

### 2.1. Search Strategy

This review was conducted according to the Preferred Reporting Items for Systematic Reviews and Meta-Analyses (PRISMA) guidelines [20] (Supplementary Table S1: PRISMA reporting checklist). PubMed, Embase, and China National Knowledge Infrastructure (CNKI) were searched up to 31 December 2021 using the following search topics: “COVID-19”, “vaccination”, “Influenza, Human”, and related terms. A time restriction filter was limited to 1 January 2019 onwards to coincide with the onset of COVID-19. The reference list of the retrieved articles were manually searched for additional articles. Our search strategies are available in Supplementary Table S2: Search strategies for PubMed, Embase and CNKI. The protocol for this systematic review was registered on PROSPERO on 25 March 2021 (ID CRD42021244193).

### 2.2. Inclusion and Exclusion Criteria

The inclusion criteria include cross-sectional studies reporting both 2019/2020 influenza vaccination uptake and 2020/2021 influenza vaccination intention and/or uptake within the same group of individuals in the study cohort. We included studies that were available in English. The exclusion criteria include studies: (1) not reporting primary data (e.g., reviews, editorial, opinion articles, and mathematical modelling studies); (2) reporting only either influenza vaccination uptake in the 19/20 pre-COVID-19 period, or vaccination uptake or intention post-COVID-19 [21,22]; (3) on effects of a programme or an activity on vaccination uptake rates [23]; (4) where sampled pre- and post-COVID populations were different, such as influenza vaccination databases [13,24]; (5) which did not report intention in 20/21 season [25]. We also excluded grey literature and articles on pre-print servers to restrict the review to high-quality, peer-reviewed studies. To prevent duplication, each study was screened for its country, date of data collection, and its database.

### 2.3. Study Selection and Data Extraction

Eligibility assessment and data extraction were carried out independently by two investigators (GK and NL) at three sequential stages: title, abstract, and full text. Discrepancies were resolved by consensus in consultation with the senior authors (YNPM and ZA). Subsequently, data were extracted from a predefined set of criteria such as author, year, hospital/database, country, study period, and participants’ demographics. Primary outcome was influenza vaccination uptake rate in 2019/2020 season and intention or uptake of influenza vaccination in the following year. Secondary outcomes included reasons behind the intention to vaccinate in the 2020/2021 influenza season, and subgroup analyses of primary outcome by region, gender, age group, and occupation.

The reported predictors of and reasons for influenza vaccination hesitancy and uptake were extracted. Data regarding the outcomes for subgroups of region, gender, age, and occupation were also extracted. Studies that reported age ranges of the participants were stratified into three groups: paediatric (≤18 years), working adults (19–50 years) and older adults (>51 years). In the paediatric age group, respondents included parents or caregivers who were responsible for making immunisation decisions for the child. As each article defined adult age groups differently, the age group with the most overlap was used as the cut-off for working adults and older adults. For example, cut-offs given in each paper which specified age groups were 45 years [26], 50 years [27,28], 55 years [29], and 60 years [30]. Each article was coded in a blinded, pairwise fashion by two investigators (GK and NL) to ensure accuracy in the coding, and discrepancies were resolved with consensus from the senior authors (YNPM and ZA).

### 2.4. Statistical Analysis and Quality Assessment

The statistical analysis of the studies was performed using Review Manager (RevMan 5.4.1), RStudio and R (R 4.0.3). A comparative analysis with Mantel-Haenszel Risk Ratios (RR) with 95% Confidence Interval (CI) was used to compare pre- and post-COVID-19 influenza vaccination. Statistical significance was established when *p*-value was <0.05. A random-effects model was used, as it is a more robust estimate regardless of heterogeneity scores [31]. Forest plots were used to present the data.

As the majority of the articles reported only vaccination intention in the 20/21 season, our main analysis included these 22 articles to reduce heterogeneity. A separate analysis was conducted for five other studies reporting the combined outcome of observed and intended uptake of vaccination in the 20/21 season. Subgroup analyses were carried out based on geographical regions, gender, age group, and occupation (healthcare workers as compared to non-healthcare workers). Subsequently, a test of heterogeneity was done using I^2^ values of 25% for mild, 50% for moderate, and 75% for high heterogeneity [32]. Lastly, systematic reporting was used to summarize the predictors and reasons for and against influenza vaccination.

Quality assessment of the studies was conducted independently using the Newcastle Ottawa Scale (NOS) adapted for cross-sectional studies by two authors (GK and NL) [33]. The NOS rates the quality of each study in three domains: selection, comparability, and outcome. Score disagreements were resolved by consensus and a final agreed-upon rating was assigned to each study where a score ≥ 7 was considered to be a high-quality study.

## 3. Results

**Figure 1:**
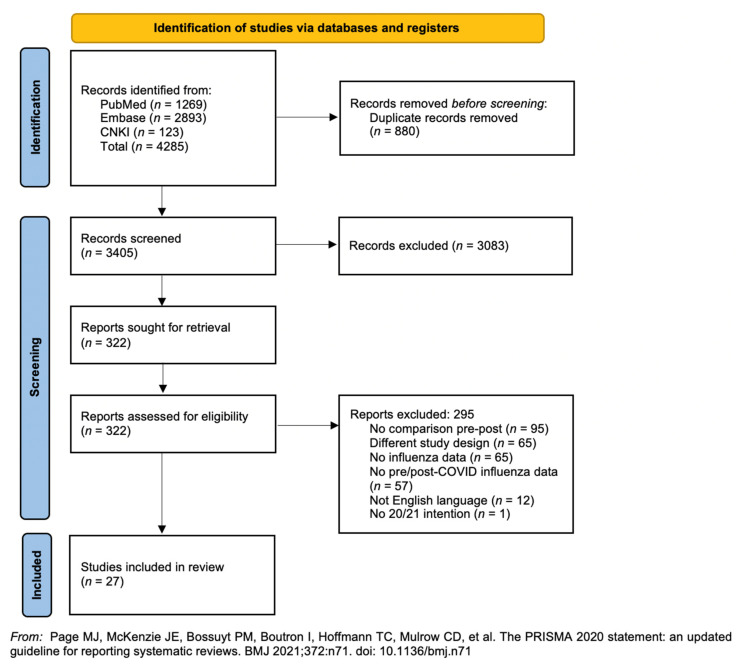
PRISMA flowchart of included studies.

The preliminary search identified 4285 articles; 3405 articles were screened after duplicates were removed. The full text of 322 articles were assessed for eligibility. Finally, 27 cross-sectional studies involving 39 193 participants were included in the meta-analysis (Figure 1). The key findings from the included studies are summarised in Table 1 [25,26,27,28,29,30,34,35,36,37,38,39,40,41,42,43,44,45,46,47,48,49,50,51,52,53,54,55].

The geographical distribution of the studies are as follows: Asia (China [44,45], Hong Kong [27], Kuwait [34], Japan [55]), Europe (France [40], Greece [46,49,54,55], Cyprus [49], Ireland [48], Italy [26,29,39,52,53], Malta [38], Poland [28], Spain [30], Turkey [43], United Kingdom [35]), and North America (United States [36,37,41,42,47,50,51]). With regards to 20/21 influenza vaccination, 22 studies reported intention and five studies reported both uptake and intention. The quality of studies based on the Newcastle-Ottawa Scale was satisfactory to good. In most of the studies, quality was limited due to self-reporting, which may be influenced by participant recall (Supplementary Table S3: Quality assessment using Newcastle-Ottawa Scale adapted for Cross-Sectional Studies).

**Figure 2:**
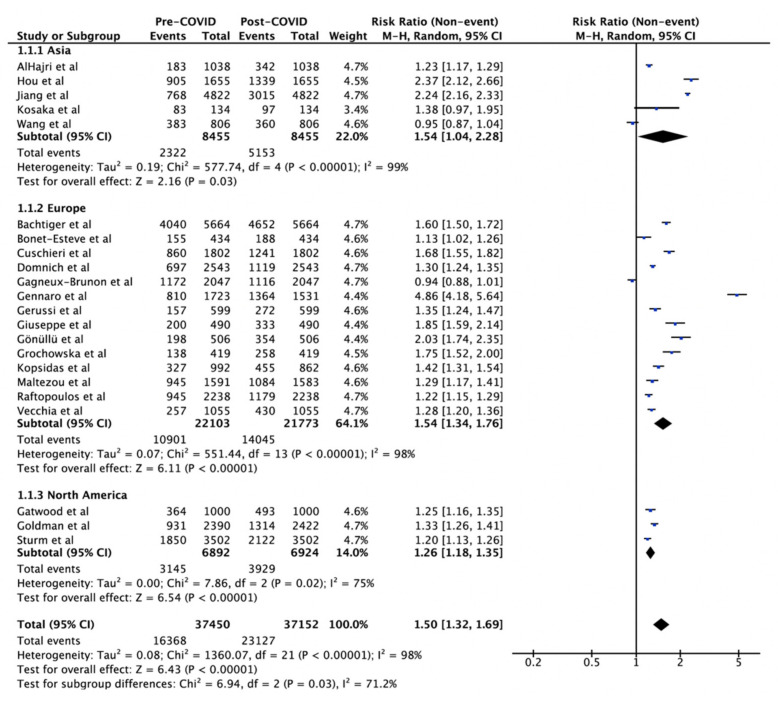
Influenza vaccination uptake pre-COVID-19 vs. intention post-COVID-19 by region. The squares and rhombus represent the individual and pooled point effect estimates with 95% confidence intervals respectively.

Overall, intention for influenza vaccination (2020/2021) post-COVID-19 was higher than in the 19/20 influenza season (RR 1.50, 95% CI 1.32–1.69, *p* < 0.001) across the 22 studies reporting intention for the 20/21 season (Figure 2). This increase in intention to vaccinate was observed in all regions: Asia (RR 1.54, 95% CI 1.04–2.28, I^2^ = 99%), Europe (RR 1.54, 95% CI 1.34–1.76, I^2^ = 98%), and North America (RR 1.26, 95% CI 1.18–1.35, I^2^ = 75%) (Figure 2). The increase in vaccination intention was significantly higher in Asia and Europe, compared to North America (*p* = 0.03) (Table 2).

**Figure 3:**
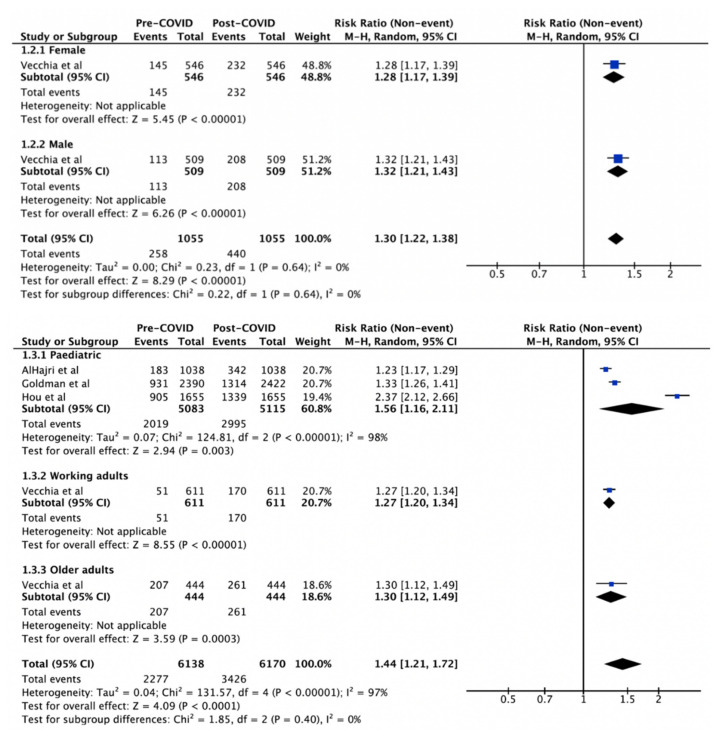
Influenza vaccination uptake pre-COVID-19 vs intention post-COVID-19 by gender and by age. The squares and rhombus represent the individual and pooled point effect estimates with 95% confidence intervals respectively.

**Figure 4:**
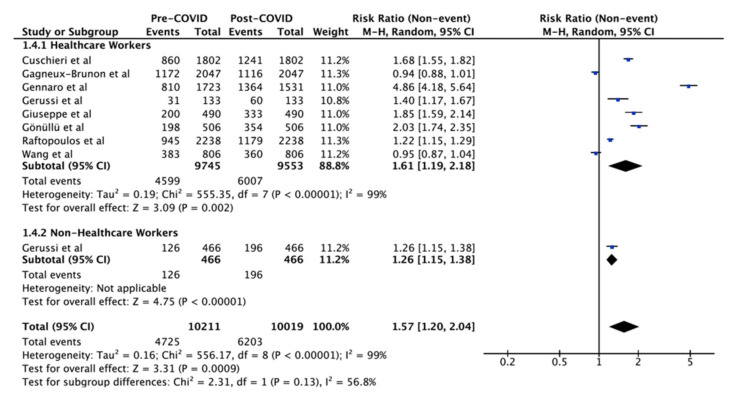
Influenza vaccination uptake pre-COVID-19 vs intention post-COVID-19 by occupation. The squares and rhombus represent the individual and pooled point effect estimates with 95% confidence intervals respectively.

The change in intention to vaccinate against influenza was investigated by gender (Figure 3), age (Figure 3), and occupation (Figure 4). All comparisons showed a significant increase in intention to vaccinate post-COVID-19. However, there was no significant difference when comparing the extent of change in intention between the genders (*p* = 0.64, I^2^ = 0%), age groups (*p* = 0.40, I^2^ = 97%), and occupation (*p* = 0.13, I^2^ = 99%) (Table 2). Significant heterogeneity was noted for all comparisons.

**Figure 5:**
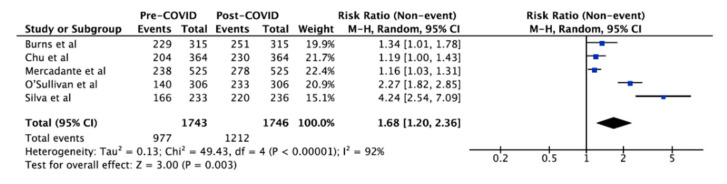
Influenza vaccination uptake pre-COVID-19 vs update and intention post-COVID-19. The squares and rhombus represent the individual and pooled point effect estimates with 95% confidence intervals respectively.

In a separate analysis of the five studies [36,37,47,48,50] that reported the combined outcome of vaccination intention and uptake in the 20/21 influenza season, there was a similar increased intention to vaccinate against influenza (RR 1.68, 95%CI 1.20–2.36) (Figure 5).

Nineteen studies reported the reasons for and against influenza vaccination for the 2020/2021 influenza season [26,27,29,30,35,36,37,39,41,42,44,45,47,48,50,51,53,54,55]. Reasons can be classified into participants’ perception of influenza vaccination, perception of influenza severity and risks, and COVID-19 pandemic and logistical issues. Participants’ perception of the vaccination included perceived efficacy of the vaccine, side effects, and fear of administration method. The main motivator for vaccination was the perceived benefits of influenza vaccination [36,37,47,48,50,53] in protecting themselves and others from influenza. Approximately 37.9–44.6% [35,47,48] of the participants felt that the vaccination could help ensure their personal and family’s safety, with one study reporting a very high rate of 71.4% [36]. However, other participants were apprehensive about taking the vaccine because they did not believe in its efficacy (e.g., they contracted influenza despite previously being vaccinated) [25,36,39,45,47,53], feared the side effects [36,37,47,53] or needles [37,39], worried about the cost [55], or believed that vaccinations are solely created to profit pharmaceutical companies [39].

The perceived risk of influenza was another driver for the intention to vaccinate. Participants who intended to vaccinate or had received the influenza 2020/21 vaccine felt that they had a higher risk of influenza illness due to their age, or concomitant health problems [35,36]. Others felt that influenza was a serious disease [36], and that it might lead to other serious health problems [36]. However, the converse beliefs were true for those who refused vaccination [36,53].

Amongst some populations, worries about COVID-19 increased influenza vaccine uptake [27,30,35,42,48,50,51,54,55]. Interestingly, one study showed that younger nurses, those working in high-risk settings, and those with higher perceived likelihood of COVID-19 infection, were more reluctant to receive influenza vaccine [27]. The authors of the study speculated that this anomalous finding might be due to the reluctance of individuals at higher risk of COVID-19 to consider vaccination for other diseases [27].

Recommendations for influenza vaccination by healthcare providers [37,41] was an important factor supporting participants’ intention for vaccination, with some individuals deferring vaccination when healthcare providers advised against vaccination [39]. Other barriers to vaccination were long wait times, distance from clinics, and time lost from work [36,37]. For some, vaccination was compulsory due to work requirements [47].

## 4. Discussion

This meta-analysis is the first to examine the effects of COVID-19 on influenza vaccination intention. The main findings of the study are: (1) increased intention to vaccinate against influenza during COVID-19 across the globe; regardless of region, age, gender, and occupation; and (2) a significant predictor of influenza vaccination intention and/or uptake was historical vaccine acceptance; other factors include individual’s perception of the severity of influenza and the safety of the vaccine.

The increased intention to vaccinate against influenza during COVID-19 is an encouraging finding, which can help mitigate negative effects of increased prevalence of coinfections [56], which has been associated with excess mortality [17,56]. As described in many of the included studies, COVID-19 pandemic was the impetus behind increased intention, indicating that the pandemic may have fostered more positive health-seeking behavior. In addition, our review found other confounding factors that contributed to improved vaccination intentions, including vaccination for personal protection and to protect others, perceived personal risk, and severity of influenza. These factors are similar to those identified in a 2011 review article, which reported threat of being at risk, worry about the disease, and social pressures to be vaccinated [57]. Evidently, these factors are important in determining intention to vaccination even prior to COVID-19. Our findings revealed that physicians can utilize the COVID-19 pandemic to boost and influence future influenza vaccination rates, especially with additional protection provided by the influenza vaccine during COVID-19 [18].

However, our review indicated that influenza vaccine hesitancy still exists. Hesitancy and mistrust in vaccines are not new findings, having been reported since the 18th century [58]. Complacency, confidence, and convenience are three important considerations to address in order to overcome vaccination hesitancy [59]. Participants who rejected vaccination due to complacency believed that influenza is not a severe disease, perceived themselves to be at low risk, and believed that influenza vaccine is of lower priority in view of the COVID-19 pandemic. The perceived low personal risk of disease was found to be an important factor in vaccine hesitancy in previous meta-analysis [60]. Some included studies demonstrated that vaccine hesitancy was also prevalent amongst healthcare professionals. This is a worrisome finding, as recommendation by healthcare professionals is a key push factor for vaccine uptake for the public.

With the implementation of public health measure such as mandatory mask wearing, many countries, such as Canada, Japan, and Singapore, reported a fall in influenza infection rates in 2020–2021 [61,62,63,64]. This information may contribute to vaccine hesitancy, as risk of influenza infection can be perceived to be lower. Furthermore, as countries begin to relax their COVID-19 restrictions while transitioning towards COVID endemicity, there may again be an increased risk of influenza transmission. In a predictive model by Lee et al. [65], influenza rates are expected to be higher in 2021–2022 season due to compensation for the light season in 2020–2021. Lee et al. suggested that improvements in either vaccine uptake or vaccine efficacy would be necessary to help avert this predicted subsequent rise in hospitalization rates.

To counter vaccine hesitancy, health campaigns and healthcare providers need to consistently emphasize vaccines as an effective way of protecting individuals and the community, as well as counter anti-vaccination messages on social media [66]. Such anti-vaccination messages were also prevalent during 2009 H1N1 pandemic [67]. Focused messages on correcting the misinformation of specific vaccines could be more effective than generic vaccination promotion campaigns [68]. These campaigns could also specifically target healthcare professionals, especially regarding misconceptions about vaccine development and safety, and mistrust against pharmaceutical companies [69].

We found that participants who had previous influenza vaccination were more likely to have increased intention to vaccinate. A review by Bish et al. suggested that interventions to improve seasonal influenza vaccination uptake among those who are currently eligible may be effective to achieve high rates of vaccination during future pandemics [57]. Because previous influenza vaccination behavior predicts intention to accept vaccination, in the face of a pandemic, it is important to promote influenza vaccination, especially to those who were previously unvaccinated.

Regarding the paediatric population, parents make the decision to vaccinate their children, weighing the benefits of protection against infections versus the potential risks and short-term distress caused by vaccination. Without parental consent, measures to improve vaccination rates among children will be limited in effectiveness. In this study, we demonstrated that there was greater intention to vaccinate children amongst caregivers who were themselves vaccinated against influenza [34]. A key step to improve vaccination rates in children would be to target their caregivers, and understand the concerns and factors that influence the caregivers’ decision [70].

Incidentally, Maltezou et al. and Gatwood et al. reported that patients who previously received influenza vaccines were also more willing to accept COVID-19 vaccines [41,46]. Evidently, improving attitudes and practices towards influenza vaccination may encourage acceptance of other vaccines, especially against diseases with significant public health impact. This could include the measles vaccine in the United States, where outbreaks have been reported due to vaccination hesitancy in recent years [71].

This meta-analysis has demonstrated that there is a positive change in intention to vaccinate against influenza, with many citing COVID-19 as an important factor in this change in attitude. Indeed, due to the nature of the studies included, our analysis was centered around the intention of a sampled population, rather than the overall observed vaccination rates. Hence, our study described the changes in vaccine attitudes and serves as a proxy for the changes in observed uptake towards influenza vaccination.

Previous studies have demonstrated that intention was a good predictor of influenza vaccination uptake among healthcare workers, with an odds ratio of 15.50 (95% CI: 9.24–25.99) [72]. Other studies have also revealed that positive intention towards vaccination was an important predictor of eventual uptake [73,74,75]. The intention-behavior gap was found to be narrower in vaccinations compared to other health behaviors [76,77,78], particularly in relation to Influenza vaccination [79].

Several emerging reports demonstrate that the COVID-19 pandemic has improved vaccine uptake rates. The Centers for Disease Control and Prevention (CDC) data from 11 jurisdictions in the United States reported that influenza vaccine administration in September-December 2020 was 9.0% higher compared to the average doses administered over the same weeks in 2018 and 2019 [80]. In addition, Fragoulis et al. reported influenza vaccination rate increased from 76% to 83% after the COVID-19 pandemic amongst patients with autoimmune rheumatic disease in a tertiary care centre in Greece [25]. Further studies are required to establish the factors affecting the translation of intention to behavior among recipients of vaccines.

This review had several limitations. There was significant heterogeneity in each of the analyses, due to variations in study and questionnaire design, as well as setting. However, we conducted subgroup analyses to mitigate this issue. Since studies were carried out at different time points (including prior to availability of the 2020/2021 influenza vaccine), the post-COVID-19 vaccination rates reported mainly intention to vaccinate, hence our study only serves as a proxy for change in vaccination behaviors. At these different time points, the severity of COVID-19 affecting the study population may vary and influence vaccine acceptance rate. Our analysis had low representation from South American, African, and Southeast Asian countries with higher risk of mortality from influenza. Despite these limitations, the consistent findings from various countries demonstrate that COVID-19 is an important motivating factor for influenza vaccination.

## 5. Conclusions

Our review highlights that COVID-19 has resulted in a more positive intention for influenza vaccination globally. However, significant hesitancy towards influenza vaccination still exists, due to low perceived risk of influenza, inefficacy, and safety concerns about vaccine. Healthcare professionals and policy makers should further encourage positive attitudes towards vaccination and focus on improving perceptions and correcting misinformation surrounding influenza and vaccination.

## Figures and Tables

**Table 1:** Summary of baseline characteristics of included studies.

Authors	Country	Study Population	Outcome Studied in 2020–2021 Season	Age (Years) *	Female	Healthcare Worker	2019/2020 Influenza Vaccine Uptake	2020/2021 Influenza Vaccine Intention and/or Uptake
Domnich et al. (2020) [39]	Italy	*n* = 2543 Italian adults	Intention	46.7 ± 15.5	45.5%	-	27.4%	44.0%
Jiang et al. (2020) [45]	China	*n* = 4822 Chinese adults	Intention	18–40: 66.1% 41–60: 30.2% >60: 3.7%	61.2%	38.0%	15.9%	62.5%
La Vecchia et al. (2020) [29]	Italy	*n* = 1055 Italian population	Intention	15–34: 23.8% 35–54: 34.1% ≥55: 42.1%	51.8%	-	24.4%	40.8%
Wang et al. (2020) [27]	Hong Kong, China	*n* = 806 Association of Hong Kong Nursing Staff	Intention	18–29: 21.6% 30–39: 31.1% 40–49: 27.1% ≥50: 20.2%	87.5%	100.0%	47.5%	44.7%
AlHajri et al. (2020) [34]	Kuwait	*n* = 1038 Kuwaiti parents and their children	Intention	<18: 100%	-	-	17.6%	32.9%
Bachtiger et al. (2020) [35]	United Kingdom	*n* = 5664 Registrants of the Care Information Exchange (CIE) of Imperial College Healthcare NHS Foundation Trust	Intention	-	50.0%	14.3%	71.3%	82.1%
Gagneux-Brunon et al. (2020) [40]	France	*n* = 2047 Healthcare workers	Intention	<30: 22.7% 30–49: 47.3% 50–64: 26.8% ≥65: 3.1%	74%	100.0%	57.3%	54.5%
Gatwood et al. (2020) [41]	United States	*n* = 1000 Tennessee adults	Intention	18–24: 17.0% 25–34: 21.7% 35–44: 24.8% 45–54: 18.1% 55–64: 18.4%	52.8%	-	36.4%	49.3%
Gerussi et al. (2021) [26]	Italy	*n* = 599 Italian patients recovered from COVID-19	Intention	53 ± 15.8	53.40%	22.2%	26.2%	45.4%
Goldman et al. (2021) [42]	US, Canada, Israel, Japan, Spain, and Switzerland	*n* = 2422 Parents and caregivers at paediatric emergency departments	Intention	8.6 ± 4.6	48.1%	-	39.0%	54.3%
Raftopoulos et al. (2021) [49]	Greece and Cypriot	*n* = 2238 Healthcare workers	Intention	Greece 40.6 ± 9.6 Rep Cyprus 35.5 ± 8.8	-	94.0%	42.2%	52.7%
Sturm et al. (2021) [51]	United States	*n* = 3502 Dynata database comprising North American survey respondents	Intention	Non-vaccinators 42.9 ± 15.2 Vaccinators 48.2 ± 17.9	51.9%	-	53.0%	60.6%
Bonet-Esteve et al. (2021) [30]	Spain	*n* = 434 Individuals registered at the Primary Care Teams of the Catalan Institute of Health of Central Catalonia	Intention	<60: 35.0% 60–70: 23.0% >70: 41.9%	59.4%	-	35.7%	43.3%
Cuschieri et al. (2021) [38]	Malta	*n* = 1802 Healthcare workers	Intention	18–24: 33.7% 25–34: 25.0% 35–44: 14.7% 45–54: 14.0% 55–64: 11.2% ≥65: 1.5%	65.2%	100.0%	48.1%	68.9%
Gönüllü et al. (2021) [43]	Turkey	*n* = 506 Turkish Paediatric Atelier	Intention	41 ± 8 26–35: 33.0% 36–44: 33.0% 45–60: 30.0% >60: 4.0%	58.0%	100.0%	39.1%	70.0%
Grochowska et al. (2021) [28]	Poland	*n* = 419 Doctors, nurses, physiotherapists, dieticians, medical students	Intention	19–25: 60.4% 26–30: 22.9% 31–40: 8.1% 41–50: 4.8% >50: 3.8%	79.0%	100.0%	32.9%	61.6%
Hou et al. (2021) [44]	China	*n* = 1655 Parents of children 3–17 years	Intention	3–5: 19.4% 6–9: 26.1% 10–14: 21.7% 15–17: 32.8%	49.9%	-	54.7%	80.9%
Maltezou et al. (2021) [46]	Greece	*n* = 1591 Healthcare workers	Intention	≤30: 17.7% 31–40: 22.8% 41–50: 28.3% >50: 31.2%	65.0%	82.7%	54%	65%
Di Gennaro et al. (2021) [52]	Italy	*n* = 1723 Healthcare workers	Intention	35.5 ± 11.8	53.0%	100.0%	47.0%	89.1%
Di Giuseppe et al. (2021) [53]	Italy	*n* = 490 Healthcare workers	Intention	50.7 ± 10.5	54.5%	100.0%	40.8%	68.0%
Kopsidas et al. (2021) [54]	Greece	*n* = 1004 Greek adult population	Intention	41.7 ± 17.7	50.2%	-	33.0%	52.8%
Kosaka et al. (2021) [55]	Japan	*n* = 163 Cancer patients	Intention	55.0 ± 12.4	60.1%	-	61.9%	72.4%
Chu et al. (2021) [37]	United States	*n* = 364 US adults above 18 years	Uptake and intention	18–29: 26.3% 30–44: 29.9% 45–60: 29.4% >60: 14.4%	59.1%	-	56.0%	63.2%
O’Sullivan et al. (2021) [48]	Ireland	*n* = 307 Patients at GP practice during the 2020 “flu season”	Uptake and intention	2–12: 13.0% 13–18: 3.9% 19–30: 13.0% 31–50: 30.3% 51–70: 29.3% >70: 9.8%	57.7%	-	45.6%	76.1%
Burns et al. (2020) [36]	United States	*n* = 315 Enrolled non-active duty patients at Landstuhl Regional Medical Center (LRMC)	Uptake and intention	21–59: 65.1% ≥60: 34.9%	42.9%	14.0%	72.7%	79.7%
Mercadante et al. (2020) [47]	United States	*n* = 525 United States adults	Uptake and intention	18–29: 21.0% 30–49: 32.8% 50–69: 32.0% ≥70: 14.1%	49.0%	-	45.3%	53.0%
Silva et al. (2021) [50]	United States	*n* = 237 US students	Uptake and intention	18–19: 43.0% 20–29: 54.0% 30–39: 2.0%	65.0%	17.0%	70.0%	93.2%

* age data presented in Mean ± SD or percentages of each age group.

**Table 2:** Comparison of influenza vaccination uptake pre-COVID-19 and intention post-COVID-19.

Comparison	Number of Studies	Sample Size	Risk Ratio (95% CI)	I^2^	*p*-Value
**Region**				98%	0.03 *
Asia	5	8455	1.54 (1.04–2.28)	99%	0.03
Europe	14	22,103/21,773	1.54 (1.34–1.76)	98%	<0.001
North America	3	6892/6924	1.26 (1.18–1.35)	75%	<0.001
**Gender**				0%	0.64 *
Female	1	546	1.28 (1.17–1.39)	-	<0.001
Male	1	509	1.32 (1.21–1.43)	-	<0.001
**Age**				97%	0.40 *
Paediatric	3	5083/5115	1.56 (1.16–2.11)	98%	0.003
Working Adults	1	611	1.27 (1.20–1.34)	-	<0.001
Older Adults	1	444	1.30 (1.12–1.49)	-	<0.001
**Occupation**				99%	0.13 *
HCW	8	9745/9553	1.61 (1.19–2.18)	99%	0.002
Non-HCW	1	466	1.26 (1.15–1.38)	-	<0.001
**Overall**	22	37,450/37,152	1.50 (1.32–1.69)	98%	<0.001

HCW—Healthcare worker. Key comparison groups are in bold font. * *p*-value for subgroup analyses of each comparison.

## Data Availability

The data for the work were derived from the published literature. This published literature is cited in the manuscript and the complete reference is provided in the Reference list.

## Supplementary Materials

The following supporting information can be downloaded at: https://www.mdpi.com/article/10.3390/vaccines10040606/s1, Table S1: PRISMA reporting checklist; Table S2: Search strategies for PubMed, Embase and CNKI; Table S3: Quality assessment using Newcastle-Ottawa Scale adapted for Cross-Sectional Studies.

## Registration

PROSPERO ID CRD 42021244193.
