# Supplementary material for: Effect of COVID-19 Pandemic on Influenza Vaccination Intention: A Meta-Analysis and Systematic Review
Source: Vaccines (Basel). 2022 Apr 13;10(4):606. doi: 10.3390/vaccines10040606 (PMC9026798; doi:10.3390/vaccines10040606)
Supplement: Supplementary file 1 [file vaccines-10-00606-s001.zip › vaccines-1633811-supplementary.pdf]

Supplementary Material

Supplementary Table S1: PRISMA reporting checklist

Supplementary Table S2: Search strategies for PubMed, Embase and CNKI

Supplementary Table S3: Quality assessment using Newcastle-Ottawa Scale adapted for Cross-Sectional Studies and References

Supplementary Table S1. PRISMA reporting checklist

Section and Topic	Item #	Checklist item	Reported on Page #
**TITLE**			
Title	1	Identify the report as a systematic review.	1
**ABSTRACT**			
Abstract	2	See the PRISMA 2020 for Abstracts checklist.	1
**INTRODUCTION**			
Rationale	3	Describe the rationale for the review in the context of existing knowledge.	1-2
Objectives	4	Provide an explicit statement of the objective(s) or question(s) the review addresses.	2
**METHODS**			
Eligibility criteria	5	Specify the inclusion and exclusion criteria for the review and how studies were grouped for the syntheses.	2
Information sources	6	Specify all databases, registers, websites, organisations, reference lists and other sources searched or consulted to identify studies. Specify the date when each source was last searched or consulted.	2-3
Search strategy	7	Present the full search strategies for all databases, registers and websites, including any filters and limits used.	Figure 1
Selection process	8	Specify the methods used to decide whether a study met the inclusion criteria of the review, including how many reviewers screened each record and each report retrieved, whether they worked independently, and if applicable, details of automation tools used in the process.	3
Data collection process	9	Specify the methods used to collect data from reports, including how many reviewers collected data from each report, whether they worked independently, any processes for obtaining or confirming data from study investigators, and if applicable, details of automation tools used in the process.	2-3
Data items	10a	List and define all outcomes for which data were sought. Specify whether all results that were compatible with each outcome domain in each study were sought (e.g. for all measures, time points, analyses), and if not, the methods used to decide which results to collect.	3
	10b	List and define all other variables for which data were sought (e.g. participant and intervention characteristics, funding sources). Describe any assumptions made about any missing or unclear information.	3
Study risk of bias assessment	11	Specify the methods used to assess risk of bias in the included studies, including details of the tool(s) used, how many reviewers assessed each study and whether they worked independently, and if applicable, details of automation tools used in the process.	3
Effect measures	12	Specify for each outcome the effect measure(s) (e.g. risk ratio, mean difference) used in the synthesis or presentation of results.	3
Synthesis methods	13a	Describe the processes used to decide which studies were eligible for each synthesis (e.g. tabulating the study intervention characteristics and comparing against the planned groups for each synthesis (item #5)).	3
	13b	Describe any methods required to prepare the data for presentation or synthesis, such as handling of missing summary statistics, or data conversions.	3
	13c	Describe any methods used to tabulate or visually display results of individual studies and syntheses.	3
	13d	Describe any methods used to synthesize results and provide a rationale for the choice(s). If meta-analysis was performed, describe the model(s), method(s) to	3

Section and Topic	Item #	Checklist item	Reported on Page #
		identify the presence and extent of statistical heterogeneity, and software package(s) used.	
	13e	Describe any methods used to explore possible causes of heterogeneity among study results (e.g. subgroup analysis, meta-regression).	3
	13f	Describe any sensitivity analyses conducted to assess robustness of the synthesized results.	3
Reporting bias assessment	14	Describe any methods used to assess risk of bias due to missing results in a synthesis (arising from reporting biases).	3
Certainty assessment	15	Describe any methods used to assess certainty (or confidence) in the body of evidence for an outcome.	3
**RESULTS**			
Study selection	16a	Describe the results of the search and selection process, from the number of records identified in the search to the number of studies included in the review, ideally using a flow diagram.	Figure 1
	16b	Cite studies that might appear to meet the inclusion criteria, but which were excluded, and explain why they were excluded.	2
Study characteristics	17	Cite each included study and present its characteristics.	Table 1
Risk of bias in studies	18	Present assessments of risk of bias for each included study.	Supplementary material 3
Results of individual studies	19	For all outcomes, present, for each study: (a) summary statistics for each group (where appropriate) and (b) an effect estimate and its precision (e.g. confidence/credible interval), ideally using structured tables or plots.	Table 2, Figure 2-4
Results of syntheses	20a	For each synthesis, briefly summarise the characteristics and risk of bias among contributing studies.	10-13
	20b	Present results of all statistical syntheses conducted. If meta-analysis was done, present for each the summary estimate and its precision (e.g. confidence/credible interval) and measures of statistical heterogeneity. If comparing groups, describe the direction of the effect.	10-13
	20c	Present results of all investigations of possible causes of heterogeneity among study results.	11
	20d	Present results of all sensitivity analyses conducted to assess the robustness of the synthesized results.	10-13
Reporting biases	21	Present assessments of risk of bias due to missing results (arising from reporting biases) for each synthesis assessed.	10
Certainty of evidence	22	Present assessments of certainty (or confidence) in the body of evidence for each outcome assessed.	13-14
**DISCUSSION**			
Discussion	23a	Provide a general interpretation of the results in the context of other evidence.	13-15
	23b	Discuss any limitations of the evidence included in the review.	18-19
	23c	Discuss any limitations of the review processes used.	18-19
	23d	Discuss implications of the results for practice, policy, and future research.	18
**OTHER INFORMATION**			
Registration and protocol	24a	Provide registration information for the review, including register name and registration number, or state that the review was not registered.	2
	24b	Indicate where the review protocol can be accessed, or state that a protocol was not prepared.	2
	24c	Describe and explain any amendments to information provided at registration or in the protocol.	-
Support	25	Describe sources of financial or non-financial support for the review, and the role of the funders or sponsors in the review.	15
Competing interests	26	Declare any competing interests of review authors.	15
Availability of data,	27	Report which of the following are publicly available and where they can be found: template data collection forms; data extracted from included studies; data	15

Section and Topic	Item #	Checklist item	Reported on Page #
code and other materials		used for all analyses; analytic code; any other materials used in the review.	

PRISMA Reporting Checklist for Abstracts

Section and Topic	Item #	Checklist item	Reported (Yes/No)
**TITLE**			
Title	1	Identify the report as a systematic review.	Yes
**BACKGROUND**			
Objectives	2	Provide an explicit statement of the main objective(s) or question(s) the review addresses.	Yes
**METHODS**			
Eligibility criteria	3	Specify the inclusion and exclusion criteria for the review.	Yes
Information sources	4	Specify the information sources (e.g. databases, registers) used to identify studies and the date when each was last searched.	Yes
Risk of bias	5	Specify the methods used to assess risk of bias in the included studies.	Yes
Synthesis of results	6	Specify the methods used to present and synthesise results.	Yes
**RESULTS**			
Included studies	7	Give the total number of included studies and participants and summarise relevant characteristics of studies.	Yes
Synthesis of results	8	Present results for main outcomes, preferably indicating the number of included studies and participants for each. If meta-analysis was done, report the summary estimate and confidence/credible interval. If comparing groups, indicate the direction of the effect (i.e. which group is favoured).	Yes
**DISCUSSION**			
Limitations of evidence	9	Provide a brief summary of the limitations of the evidence included in the review (e.g. study risk of bias, inconsistency and imprecision).	Yes
Interpretation	10	Provide a general interpretation of the results and important implications.	Yes
**OTHER**			
Funding	11	Specify the primary source of funding for the review.	Yes
Registration	12	Provide the register name and registration number.	Yes

From: Page MJ, McKenzie JE, Bossuyt PM, Boutron I, Hoffmann TC, Mulrow

Supplementary Table S2: Search Strategy

PubMed

#1	“SARS-CoV-2"[Mesh] OR "COVID-19"[Mesh] OR "Coronavirus Infections"[MeSH Terms] OR 2019-nCoV[Title/Abstract] OR 2019 ncov[Title/Abstract] OR nCov[Title/Abstract] OR covid-19[Title/Abstract] OR covid19[Title/Abstract] OR COVID-2019[Title/Abstract] OR COVID2019[Title/Abstract] OR sars-cov-2[Title/Abstract] OR "sars cov-2"[Title/Abstract] OR sarscov2[Title/Abstract] OR sarscov-2[Title/Abstract] OR sars-coronavirus-2[Title/Abstract] OR sars corona virus[Title/Abstract] OR sars-like coronavirus[Title/Abstract] OR novel coronavirus[Title/Abstract] OR novel corona virus[Title/Abstract] OR covid*[Title/Abstract] OR coronavirus 2[Title/Abstract] OR coronavirus infection*[Title/Abstract] OR coronavirus disease[Title/Abstract] OR corona virus disease[Title/Abstract] OR new coronavirus[Title/Abstract] OR new corona virus[Title/Abstract] OR new coronaviruses[Title/Abstract] OR novel coronaviruses[Title/Abstract] OR severe acute respiratory syndrome coronavirus-2[Title/Abstract] OR ((coronavirus[Title/Abstract] OR ncov[Title/Abstract] OR sars-cov[Title/Abstract]) AND (2019[Title/Abstract] OR 19[Title/Abstract] OR Wuhan[Title/Abstract]))
#2	(“Influenza, Human"[Mesh]) OR (influenza*[Title/Abstract] OR flu[Title/Abstract] OR H5n1[Title/Abstract] OR H1n1[Title/Abstract])
#3	(“Vaccination"[Mesh]) OR ("vaccin*"[Title/Abstract] OR “inoculat*"[Title/Abstract] OR Immuniz*[Title/Abstract] OR Immunis*[Title/Abstract])
#4	#1 AND #2 AND #3 AND ("2020/1/1"[Date - Publication] : "3000"[Date - Publication])

Embase

#1	'covid 19'/exp OR 'coronavirus disease 2019'/exp OR 'coronavirus infection'/exp OR 'severe acute respiratory syndrome coronavirus 2'/exp OR '2019-ncov':ti,ab OR '2019 ncov':ti,ab OR ncov:ti,ab OR 'covid-19':ti,ab OR 'covid-2019':ti,ab OR 'covid2019':ti,ab OR covid19:ti,ab OR 'sars-cov-2':ti,ab OR 'sars cov-2':ti,ab OR sarscov2:ti,ab OR 'sarscov-2':ti,ab OR 'sars-coronavirus-2':ti,ab OR 'sars corona virus':ti,ab OR 'sars-like coronavirus':ti,ab OR 'novel coronavirus':ti,ab OR 'novel corona virus':ti,ab OR covid*:ti,ab OR 'coronavirus 2':ti,ab OR 'coronavirus infection*':ti,ab OR 'coronavirus disease':ti,ab OR 'corona virus disease':ti,ab OR 'new coronavirus':ti,ab OR 'new corona virus':ti,ab OR 'new coronaviruses':ti,ab OR 'novel coronaviruses':ti,ab OR 'severe acute respiratory syndrome coronavirus-2':ti,ab OR ((coronavirus:ti,ab OR ncov:ti,ab OR 'sars cov':ti,ab) AND (2019:ti,ab OR 19:ti,ab OR wuhan:ti,ab))
#2	'vaccination' OR 'influenza vaccination' OR 'virus vaccine' OR 'inoculation' OR 'vaccination'/exp OR immunis*:ab,ti OR immuniz*:ab,ti OR vaccin*:ab,ti OR inoculat*:ab,ti
#3	'influenza' OR 'influenza'/exp OR influenza*:ab,ti OR h5n1:ab,ti OR h1n1:ab,ti OR flu:ab,ti
#4	#1 AND #2 AND #3 AND [1-1-2020]/sd

CNKI

#1	COVID-19
#2	Influenza
#3	Vaccination
#4	#1 and #2 and #3
#5	#4 AND Publication date: 2020-01-01 onwards

Supplementary Table S3. Quality assessment using Newcastle-Ottawa Scale adapted for Cross-Sectional Studies.

	Study (first author)	**Selection: (Maximum 5 stars)**				**Comparability: (Maximum 2 stars)**	**Outcome: (Maximum 3 stars)**		**Total Score**
		1. Representativeness of the sample: a. Truly representative of the average in the target population. * (all subjects or random sampling) b. Somewhat representative of the average in the target group. * (non-random sampling) c. Selected group of users/convenience sample. d. No description of the derivation of the included subjects.	2. Sample size: a. Justified and satisfactory (including sample size calculation). * b. Not justified. c. No information provided	3. Non-respondents: a. Proportion of target sample recruited attains pre-specified target or basic summary of non-respondent characteristics in sampling frame recorded. * b. Unsatisfactory recruitment rate, no summary data on non-respondents. c. No information provided .	4. Ascertainment of the exposure (risk factor): a. Vaccine records/vaccine registry/clinic registers/hospital records only. ** b. Parental or personal recall and vaccine/hospital records. * c. Parental/personal recall only.	1. Comparability of subjects in different outcome groups on the basis of design or analysis. Confounding factors controlled. a. Data/ results adjusted for relevant predictors/risk factors/confounders e.g. age, sex, time since vaccination, etc. ** b. Data/results not adjusted for all relevant confounders/risk factors/information not provided.	1. Assessment of outcome: a. Independent blind assessment using objective validated laboratory methods. ** b. Unblinded assessment using objective validated laboratory methods. ** c. Self report. * d. No description/non-standard laboratory methods used.	2. Statistical test: a. Statistical test used to analyse the data clearly described, appropriate and measures of association presented including confidence intervals and probability level (p value). * b. Statistical test not appropriate, not described or incomplete.	
1	*Domnich et al*[1]	*	*	*		**	*	*	7
2	*Jiang et al*[2]					**	*	*	4
3	*La Vecchia et al*[3]	*					*		2
4	*Mercadante et al*[4]	*				**	*	*	5
5	*Wang et al*[5]	*	*	*		**	*	*	7
6	*AlHajri et al*[6]						*	*	2
7	*Bachtiger et al*[7]	*				**	*	*	5
8	*Burns et al*[8]	*	*	*		**	*	*	7
9	*Silva et al*[9]	*		*		**	*	*	6
10	*Gagneux-Brunon et al*[10]	*				**	*	*	5
11	*Gatwood et al*[11]	*	*	*		**	*	*	7

12	*Gerussi et al*[12]	*		*		**	*	*	6
13	*Goldman et al*[13]	*		*		**	*	*	6
14	*Raftopoulos et al*[14]					**	*	*	4
15	*Sturm et al*[15]	*		*		**	*	*	6
16	*Chu et al*[16]	*	*	*		**	*	*	7
17	*O'Sullivan et al*[17]						*	*	2
18	*Bonet-Esteve et al*[18]	*	*	*		**	*	*	7
19	*Cuschieri et al*[19]	*					*	*	3
20	*Gönüllü et al*[20]	*				**	*	*	5
21	*Grochowska et al*[21]	*				**	*	*	5
22	*Hou et al*[22]	*	*			**	*	*	6
23	*Maltezou et al*[23]	*				**	*	*	5
24	*Giuseppe et al*[24]	*	*	*		**	*	*	7
25	*Gennaro et al*[25]			*		**	*	*	5
26	*Kopsidas et al*[26]	*		*		**	*	*	6
27	*Kosaka et al*[27]			*		**	*	*	5

Legend for cross-sectional studies:

Very Good Studies	9-10 points
Good Studies	7-8 points
Satisfactory Studies	5-6 points
Unsatisfactory Studies	0 to 4 points

References

1. Domnich, A.; Cambiaggi, M.; Vasco, A.; Maraniello, L.; Ansaldi, F.; Baldo, V.; Bonanni, P.; Calabrò, G.E.; Costantino, C.; de Waure, C. Attitudes and beliefs on influenza vaccination during the COVID-19 pandemic: results from a representative Italian survey. *Vaccines* **2020**, *8*, 711.
2. Jiang, M.; Feng, L.; Wang, W.; Gong, Y.; Ming, W.-K.; Hayat, K.; Li, P.; Gillani, A.H.; Yao, X.; Fang, Y. Knowledge, attitudes, and practices towards influenza among Chinese adults during the epidemic of COVID-19: a cross-sectional online survey. *Hum Vaccin Immunother* **2021**, *17*, 1412–1419.
3. La Vecchia, C.; Negri, E.; Alicandro, G.; Scarpino, V. Attitudes towards influenza vaccine and a potential COVID-19 vaccine in Italy and differences across occupational groups, September 2020. *Med Lav* **2020**, *111*, 445–448.
4. Mercadante, A.R.; Law, A.V. Will they, or Won't they? Examining patients' vaccine intention for flu and COVID-19 using the Health Belief Model. *Res Social Adm Pharm* **2020**, *17*, 1596–1605.
5. Wang, K.; Wong, E.L.Y.; Ho, K.F.; Cheung, A.W.L.; Chan, E.Y.Y.; Yeoh, E.K.; Wong, S.Y.S. Intention of nurses to accept coronavirus disease 2019 vaccination and change of intention to accept seasonal influenza vaccination during the coronavirus disease 2019 pandemic: A cross-sectional survey. *Vaccine* **2020**, *38*, 7049–7056.
6. AlHajri, B.; Alenezi, D.; Alfouzan, H.; Altamimi, S.; Alzalzalah, S.; Almansouri, W.; Alqudeimat, Y.; Almokhaizeem, Z.; Ziyab, A.H. Willingness of parents to vaccinate their children against influenza and the novel coronavirus disease-2019. *J Pediatr* **2021**, *231*, 298–299.
7. Bachtiger, P.; Adamson, A.; Chow, J.-J.; Sisodia, R.; Quint, J.K.; Peters, N.S. The impact of the COVID-19 pandemic on the uptake of influenza vaccine: UK-wide observational study. *JMIR Public Health Surveill* **2021**, *7*, e26734.
8. Burns, V.M.; Castillo, F.M.; Coldren, C.R.L.; Prosser, T.; Howell, L.P.R.L.; Kabbur, M.B. Perceptions of seasonal influenza vaccine among US Army civilians and dependents in the Kaiserslautern Military Community: a mixed-methods survey. *Mil Med* **2021**, usaa572.
9. Silva, J.; Bratberg, J.; Lemay, V. COVID-19 and influenza vaccine hesitancy among college students. *J Am Pharm Assoc (2003)* **2021**, S1544-3191(1521)00191-00196.
10. Gagneux-Brunon, A.; Detoc, M.; Bruel, S.; Tardy, B.; Rozaire, O.; Frappe, P.; Botelho-Nevers, E. Intention to get vaccinations against COVID-19 in French healthcare workers during the first pandemic wave: a cross-sectional survey. *J Hosp Infect* **2021**, *108*, 168–173.
11. Gatwood, J.; McKnight, M.; Fiscus, M.; Hohmeier, K.C.; Chisholm-Burns, M. Factors influencing likelihood of COVID-19 vaccination: A survey of Tennessee adults. *Am J Health Syst Pharm* **2021**, *78*, 879–889.
12. Gerussi, V.; Peghin, M.; Palese, A.; Bressan, V.; Visintini, E.; Bontempo, G.; Graziano, E.; De Martino, M.; Isola, M.; Tascini, C. Vaccine hesitancy among Italian patients recovered from COVID-19 infection towards influenza and Sars-Cov-2 vaccination. *Vaccines* **2021**, *9*, 172.
13. Goldman, R.D.; McGregor, S.; Marneni, S.R.; Katsuta, T.; Griffiths, M.A.; Hall, J.E.; Seiler, M.; Klein, E.J.; Cotanda, C.P.; Gelernter, R. Willingness to vaccinate children against influenza after the Coronavirus disease 2019 pandemic. *J Pediatr* **2021**, *228*, 87–93. e82.
14. Raftopoulos, V.; Iordanou, S.; Katsapi, A.; Dedoukou, X.; Maltezou, H.C. A comparative online survey on the intention to get COVID-19 vaccine between Greek and Cypriot healthcare personnel: is the country a predictor? *Hum Vaccin Immunother* **2021**, 1–8.
15. Sturm, L.; Kasting, M.L.; Head, K.J.; Hartsock, J.A.; Zimet, G.D. Influenza vaccination in the time of COVID-19: A national US survey of adults. *Vaccine* **2021**, *39*, 1921–1928.
16. Chu, A.; Gupta, V.; Unni, E.J. Utilizing the Theory of Planned Behavior to determine the intentions to receive the influenza vaccine during COVID-19: A cross-sectional survey of US adults. *Prev Med Rep* **2021**, 101417.
17. O'Sullivan, N.; O'Sullivan, G.; Van Harten, M. The Impact of the COVID-19 Pandemic on the Uptake of the Seasonal Influenza Vaccine. *Ir Med J* **2021**.
18. Bonet-Esteve, A.; Muñoz-Miralles, R.; Gonzalez-Claramunt, C.; Rufas, A.M.; Cruz, X.P.; Vidal-Alaball, J. Influenza vaccination during the coronavirus pandemic: intention to vaccinate among the at-risk population in the Central Catalonia Health Region (VAGCOVID). *BMC Fam Pract* **2021**, *22*, 1–9.
19. Cuschieri, S.; Grech, V. A comparative assessment of attitudes and hesitancy for influenza vis-à-vis COVID-19 vaccination among healthcare students and professionals in Malta. *J Public Health* **2021**, 1-8.

20. Gönüllü, E.; Soysal, A.; Atıcı, S.; Engin, M.; Yeşilbaş, O.; Kasap, T.; Fedakar, A.; Bilgiç, E.; Tavil, E.B.; Tutak, E. Pediatricians' COVID-19 experiences and views on the willingness to receive COVID-19 vaccines: a cross-sectional survey in Turkey. *Hum Vaccin Immunother* **2021**, 1–8.
21. Grochowska, M.; Ratajczak, A.; Zdunek, G.; Adamiec, A.; Waszkiewicz, P.; Feleszko, W. A Comparison of the Level of Acceptance and Hesitancy towards the Influenza Vaccine and the Forthcoming COVID-19 Vaccine in the Medical Community. *Vaccines* **2021**, *9*, 475.
22. Hou, Z.; Song, S.; Du, F.; Shi, L.; Zhang, D.; Lin, L.; Yu, H. The Influence of the COVID-19 Epidemic on Prevention and Vaccination Behaviors Among Chinese Children and Adolescents: Cross-sectional Online Survey Study. *JMIR Public Health Surveill* **2021**, *7*, e26372.
23. Maltezou, H.C.; Pavli, A.; Dedoukou, X.; Georgakopoulou, T.; Raftopoulos, V.; Drositis, I.; Bolikas, E.; Ledda, C.; Adamis, G.; Spyrou, A. Determinants of intention to get vaccinated against COVID-19 among healthcare personnel in hospitals in Greece. *Infect Dis Health* **2021**, *26* 189–197.
24. Di Giuseppe, G.; Pelullo, C.P.; Paolantonio, A.; Della Polla, G.; Pavia, M. Healthcare Workers' Willingness to Receive Influenza Vaccination in the Context of the COVID-19 Pandemic: A Survey in Southern Italy. *Vaccines (Basel)* **2021**, *9*, doi:10.3390/vaccines9070766.
25. Di Gennaro, F.; Murri, R.; Segala, F.V.; Cerruti, L.; Abdulle, A.; Saracino, A.; Bavaro, D.F.; Fantoni, M. Attitudes towards anti-sars-cov2 vaccination among healthcare workers: Results from a national survey in Italy. *Viruses* **2021**, *13*, doi:10.3390/v13030371.
26. Kopsidas, I.; Chorianopoulou, E.; Kourkouni, E.; Triantafyllou, C.; Molocha, N.-M.; Koniordou, M.; Maistreli, S.; Tsopela, C.G.; Maroudi-Manta, S.; Filippou, D.; et al. COVID-19 pandemic impact on seasonal flu vaccination: A cross-sectional study. *Pneumon* **2021**, *34*, 1-9, doi:10.18332/pne/136173.
27. Kosaka, M.; Kotera, Y.; Tsuda, K.; Takahashi, K.; Hamaki, T.; Kusumi, E.; Kami, M.; Tanimoto, T. Influenza vaccination uptake and attitudes among adult cancer patients in Japan: a web-based questionnaire survey before the 2020/2021 season. *Hum Vaccin Immunother* **2021**, *17*, 5509-5513, doi:10.1080/21645515.2021.1977569.
